# New Delhi Metallo-β-Lactamase-5 (NDM-5)-Producing Klebsiella pneumoniae Urinary Tract Infection in a Young Patient Without a History of Overseas Travel: A Case Suggesting Local Spread in Japan

**DOI:** 10.7759/cureus.115335

**Published:** 2026-08-28

**Authors:** Nao Nishimura, Hideyuki Hayashi, Makoto Kuroda, Tatsuya Kawaguchi, Hirotomo Nakata, Jun-Ichirou Yasunaga

**Affiliations:** 1 Department of Hematology, Rheumatology and Infectious Diseases, Kumamoto University Hospital, Kumamoto, JPN; 2 Department of Clinical Laboratory, Kumamoto University Hospital, Kumamoto, JPN; 3 Department of Medical Technology, Kumamoto Health Science University, Kumamoto, JPN

**Keywords:** carbapenemase-producing enterobacterales, klebsiella pneumoniae, ndm-5, sequence type 290, whole-genome sequencing

## Abstract

We isolated New Delhi metallo-β-lactamase-5 (NDM-5)-producing *Klebsiella pneumoniae* KU-00156 from a young individual with no history of overseas travel or exposure to foreign healthcare facilities. During hematologic recovery after chemotherapy for osteosarcoma, the patient developed a urinary tract infection. Urine cultures yielded *Klebsiella pneumoniae* and *Enterococcus faecalis*. The *K. pneumoniae* isolate demonstrated resistance to multiple β-lactam antibiotics, including carbapenems, and PCR-based carbapenemase gene testing confirmed the presence of NDM. Whole-genome analysis revealed that the strain KU-00156 was an NDM-5-producing *K. pneumoniae *ST290, harboring multiple resistance genes, including *bla*_NDM-5_, on an IncHI2-replicon type plasmid. This case represents the detection of NDM-5-producing *K. pneumoniae* from a young individual without a history of overseas exposure and is considered an important case suggesting the possible prevalence of NDM-5 in the Japanese community.

## Introduction

Carbapenemase-producing Enterobacterales (CPE) represent a major global public health threat. Among the carbapenemases, New Delhi metallo-β-lactamase (NDM) is a class B metallo-β-lactamase that hydrolyzes nearly all β-lactam antibiotics except monobactams and has spread worldwide [1-3]. NDM-5, first described in 2011, differs from NDM-1 by two amino acid substitutions (V88L and M154L), resulting in enhanced carbapenem-hydrolyzing activity [4,5].

The molecular epidemiology of CPE varies geographically. In Japan, IMP-type carbapenemases have historically predominated, whereas KPC-type carbapenemases are prevalent in North America, OXA-48-like enzymes in Europe and the Middle East, and NDM-type enzymes in South Asia and East Asia [6-8]. Recent nationwide surveillance has demonstrated an increase in non-IMP carbapenemases, including NDM-producing Enterobacterales, in Japan due to the increase in international travel and trade [7-9].

NDM-5-producing Enterobacterales have become increasingly prevalent in East Asia, particularly in China, South Korea, and Taiwan, and have been associated with the dissemination of transferable plasmids such as IncX3 [10-13]. Although sporadic cases have also been reported in Japan, information regarding the molecular characteristics and epidemiology of NDM-5-producing Klebsiella pneumoniae remains limited.

Here, we report a urinary tract infection caused by NDM-5-producing Klebsiella pneumoniae ST290 isolated from a young patient without a history of overseas travel or exposure to foreign healthcare facilities. We further characterized the isolate by whole-genome sequencing to investigate its molecular features and discuss its epidemiological implications.

## Case presentation

The patient was a male in his teens with no history of overseas travel. He was admitted for chemotherapy for osteosarcoma localized to the mandible. He was undergoing his third cycle of vincristine, doxorubicin, and cyclophosphamide, alternating with ifosfamide and etoposide (VDC/IE) chemotherapy. No therapeutic antibiotics had been administered during the previous chemotherapy cycles, except for prophylactic trimethoprim-sulfamethoxazole (TMP-SMX). Six days after completing the IE cycle, during hematological recovery from chemotherapy-induced myelosuppression, he developed fever and lower abdominal pain. Laboratory findings on presentation showed a white blood cell count of 7,900/μL (81% neutrophils) and a C-reactive protein (CRP) level of 5.72 mg/dL (Table 1).

**Table 1:** Laboratory findings on the date of onset WBC: white blood cell count; Hb: hemoglobin; Plt: platelet count; Alb: albumin; T-Bil: total bilirubin; AST: aspartate aminotransferase; ALT: Alanine aminotransferase; Crea: creatinine; CRP: C-reactive protein; PCT: procalcitonin

Parameter	Value	Unit	Reference range
WBC	7900	/µL	3,300-8,600
Neutrophils	81	%	42.4-75.0
Lymphocytes	5	%	18.2-47.7
Monocytes	12	%	3.3-9.0
Hb	10.4	g/dL	11.6-14.8
Plt	22.5 × 10^4^	/µL	15.8-34.8 × 10^4^
Alb	4	g/dL	4.1-5.1
T-Bil	0.5	mg/dL	0.4-1.5
AST	38	U/L	13-30
ALT	36	U/L	10-42
Crea	0.71	mg/dL	0.46-0.79
CRP	5.72	mg/dL	0.00-0.14
PCT	0.12	ng/ml	<0.05

The hemodynamics were stable, and no findings suggested sepsis.

A urinary tract infection was suspected. As the patient did not have an indwelling urinary catheter, urine and blood cultures were obtained before initiating treatment with levofloxacin (LVFX). LVFX was administered for six days. He became afebrile within two days of initiating treatment, and his symptoms gradually resolved.

Blood cultures were negative. Quantitative urine culture revealed a Gram-negative bacillus (10⁴ CFU/mL) and Gram-positive coccus (10³ CFU/mL). After incubation on sheep blood agar and BTB lactose agar, the isolates were identified as *Klebsiella pneumoniae *and* Enterococcus faecalis*, respectively, using matrix-assisted laser desorption/ionization time-of-flight mass spectrometry (MALDI-TOF MS; VITEK MS, bioMérieux, Marcy-l'Étoile, France). Antimicrobial susceptibility testing was performed using a DPS192iX microbial susceptibility analyzer and a dedicated EP31 panel (Eiken Chemical). To measure the minimum inhibitory concentration (MIC) of cefiderocol, the Shionogi MIC Dry Plate Cefiderocol (manufacturer: Kyokuto Pharmaceutical Industrial, distributor: Shionogi & Co., Ltd.) was used. In antimicrobial susceptibility testing of *K. pneumoniae* (strain KU-00156), high resistance to cephalosporins, penicillins, and carbapenem antibiotics was observed. The MICs of both imipenem and meropenem were > 8 μg/mL. Although cefiderocol retained its activity, it was at the upper limit of the susceptibility breakpoint. In contrast, the susceptibility to amikacin, minocycline and LVFX was maintained (Table 2) [14].

**Table 2:** Antimicrobial susceptibility profile of Klebsiella pneumoniae MIC breakpoints were based on the Clinical and Laboratory Standards Institute (CLSI) M100, 36th edition. "–" indicates that no CLSI interpretive criteria are available. * indicates SDD (susceptible dose-dependent). MIC: minimum inhibitory concentration

Antibiotics	MIC (ug/ml)	MIC breakpoints		
		S	I	R
Ampicillin	>16	≤8	16	≥32
Piperacillin/Tazobactam	>64	≤8/4	16/4	≥32/4
Cefazolin	>16	≤2	4	≥8
Cefotiam	>4	-	-	-
Ceftriaxone	>32	≤1	2	≥4
Ceftazidime	>16	≤4	8	≥16
Cefepime	16	≤2	4–8*	≥16
Cefmetazole	16	16	32	64
Aztreonam	<0.5	≤4	8	≥16
Imipenem	>8	≤1	2	≥4
Meropenem	>8	≤1	2	≥4
Amikacin	<4	≤4	8	≥16
Minocycline	4	≤4	8	≥16
Levofloxacin	0.5	≤0.5	1	≥2
Ciprofloxacin	0.5	≤0.25	0.5	≥1
Fosfomycin	128	-	-	-
Cefiderocol	4	≤4	8	≥16

A follow-up urine culture obtained three weeks after completion of antimicrobial therapy yielded only *Enterococcus *species at 10² CFU/mL, with no growth of *K. pneumoniae*. Although *E. faecalis* was co-isolated with *K. pneumoniae*, the patient's clinical presentation, prompt response to levofloxacin, and the absence of *K. pneumoniae* on follow-up urine culture suggested that *K. pneumoniae* was the primary pathogen.

Suspecting a carbapenemase-producing organism, we tested for carbapenemase genes using the GeneXpert System and detected the NDM gene.

DNA sequencing libraries were prepared using QIAseq FX (QIAGEN) and sequenced using the iSeq 100 sequencer (Illumina, San Diego, CA, USA) with 150 bp paired-end reads. Additionally, a long-read sequencing library was prepared using the Ligation Sequencing gDNA-Native Barcoding Kit 24 V14 (Oxford Nanopore Technologies, Oxford, UK), followed by sequencing on a MinION Mk1b using an R10.4.1 flow cell controlled by MinKNOW v25.05.14, with basecalling carried out using Dorado v7.9.8. Hybrid assembly of the Illumina and Nanopore reads using Unicycler v0.4.8 yielded a circular complete genome sequence of a chromosome and plasmids.

Genome annotations were performed using DFAST v1.6.0 [15] and ResFinder 4.7.2 [16]. The circular map of genome annotation was visualized using the Proksee website [17].

**Figure 1:**
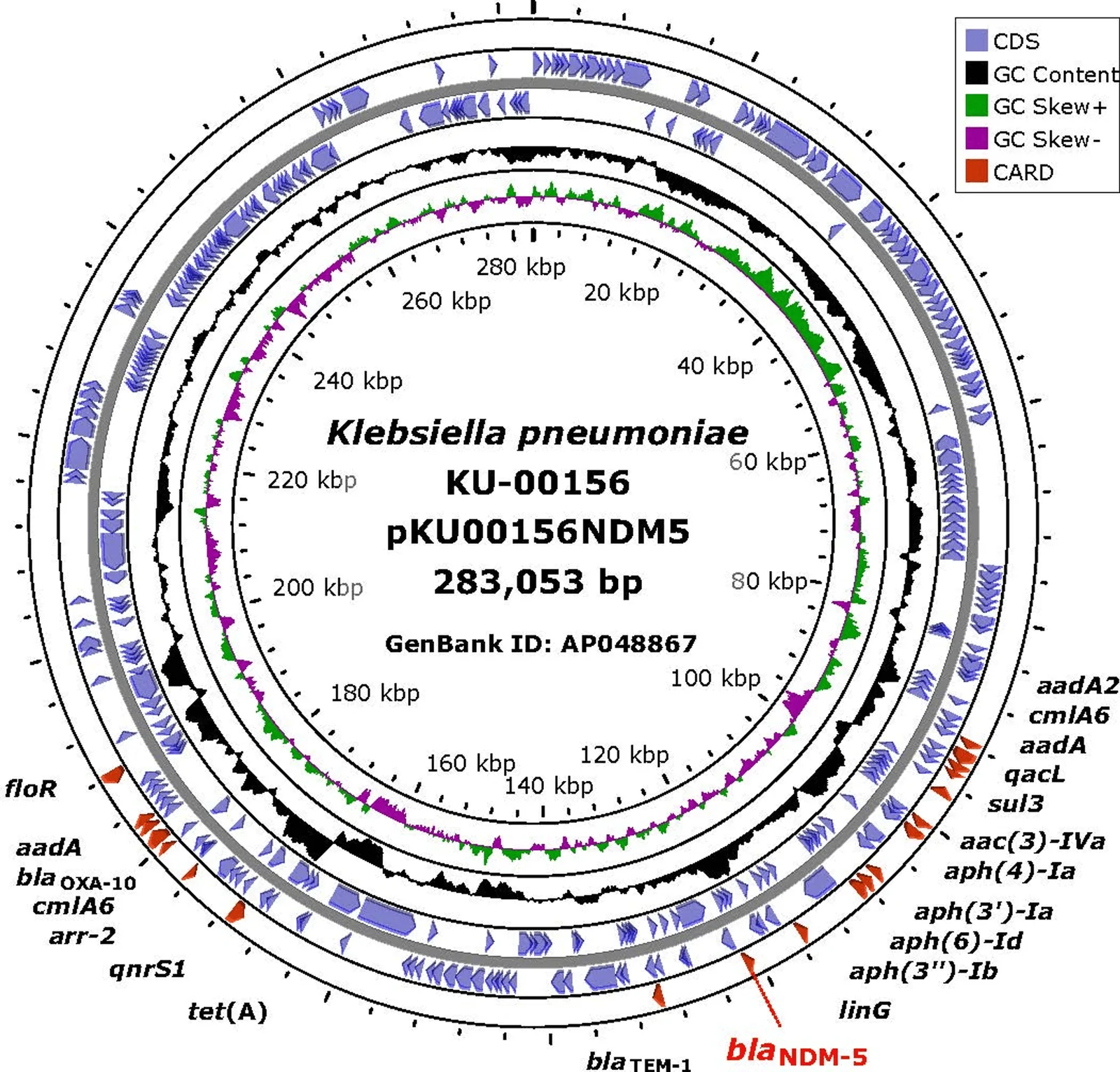
Plasmid map of KU-00156 The circular plasmid map representation of NDM-5-positive pKU00156NDM5 was illustrated by the Proksee website [17]. Starting from the outermost ring, ring 1: antimicrobial resistance genes identified by The Comprehensive Antibiotic Resistance Database [18]; ring2: + stranded coding sequence (CDS) features: backbone DNA sequence; ring4: - stranded coding sequence (CDS) features (- strand); ring 5: GC content; ring 6: GC skew. Antimicrobial resistance genes were highlighted outside the rings.

It suggested that KU-00156 was classified as ST 290 in the Klebsiella PasteurMLST database and possessed an IncHI2 plasmid pKU00156NDM5 (283,053 bp) carrying blaNDM-5 and other antimicrobial resistance genes [aadA22, aac(3)-IV, aph(3'')-Ib, blaTEM-1B, blaOXA-10, qnrS1, sul3, tet(A), and floR[MK1]] (Figure 1).

The circular map of plasmid annotation was visualized using the Proksee website [19].

**Figure 2:**
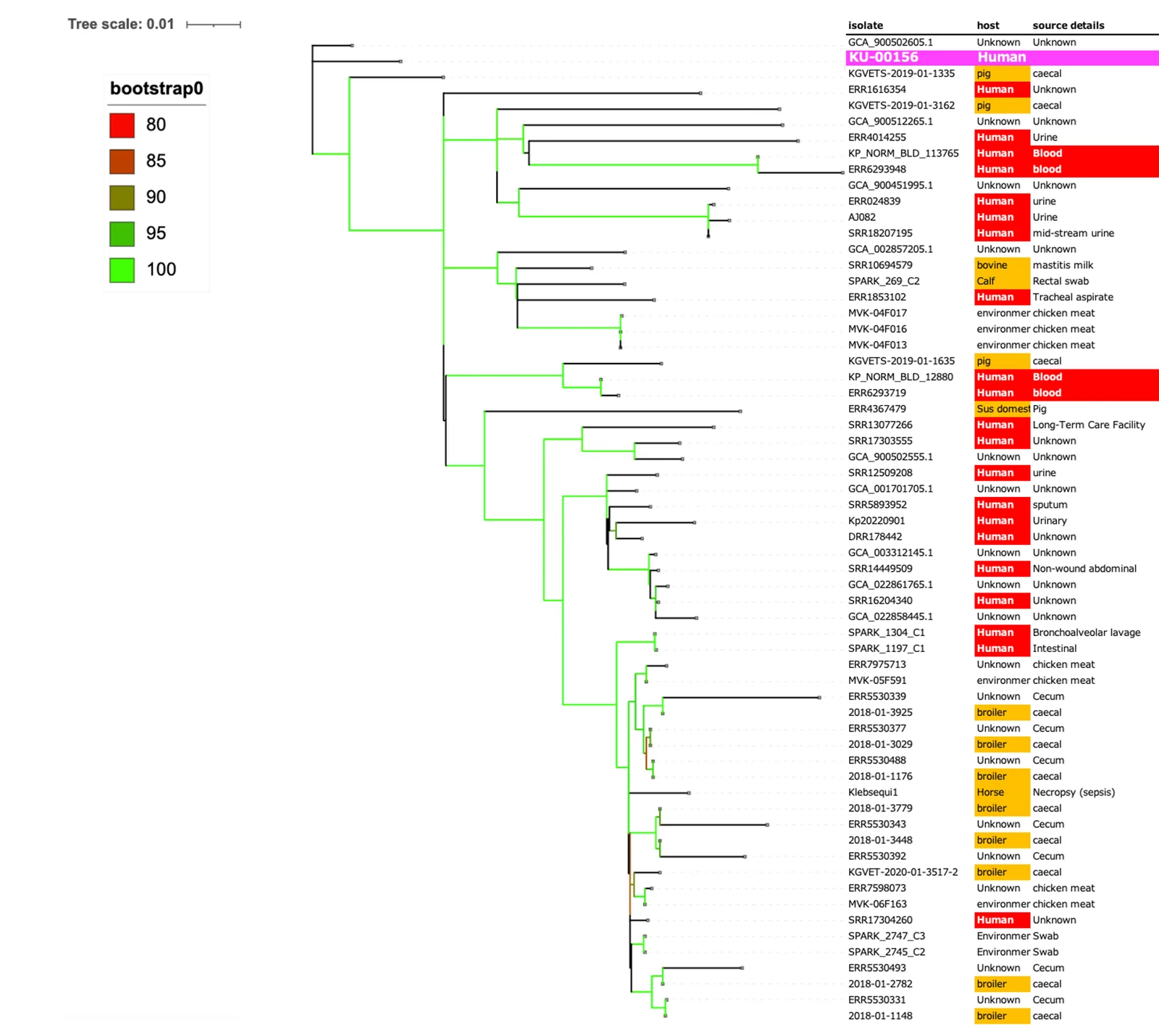
Core genome single-nucleotide variation phylogenetic analysis of K. pneumoniae ST290 strains The phylogenetic tree with 1000-fold bootstrap replicates (bootstrap values are shown at each branch).

We collected the genomic information of 61 *K. pneumoniae* ST290 strains registered in PubMLST hosted by the Institut Pasteur MLST Databases (https://bigsdb.pasteur.fr/klebsiella/) and performed core genome phylogenetic trees; pairwise single-nucleotide variants were determined using Parsnp v1.7.4. Phylogenetic relationships were evaluated using iQtree version 2.2.5 with 1000-fold bootstrap replicates using the following parameters (iqtree2-s parsnp.snps.mblocks-alrt 1000-B 1000-nt 6). The phylogenetic tree was visualized using the iTOL website (Figure 2) [20]. No significant strains were obtained, indicating that KU-00156 is a unique hypervirulent clone.

## Discussion

In the present case, NDM-5-producing *K. pneumoniae* ST290 was detected in a teenage boy with no history of overseas travel or exposure to foreign medical institutions. The molecular epidemiology of CPE in Japan has long been characterized by the predominance of IMP-type carbapenemases, whereas NDM-type carbapenemases remain relatively uncommon [7]. However, recent national surveillance has demonstrated an increase in non-IMP carbapenemases, including NDM, KPC, and OXA-48-like-producing Enterobacterales [9]. These findings suggest that the molecular epidemiology of CPE in Japan may be changing. Most cases of NDM-5 reported in Japan have been infections caused by Escherichia coli, with the majority of patients being elderly individuals in their 70s-90s years of age. Urinary tract infections and bacteremia are common manifestations of these infections. Although some cases have been reported without a history of overseas travel, many have been associated with medical institutions and nursing facilities [21,22]. In contrast, the present case is notable for occurring in a young individual and involving *K. pneumoniae* rather than *E. coli*, which has been the predominant host species among NDM-5-producing Enterobacterales reported in Japan. Unlike globally disseminated high-risk clones such as ST11, ST15, ST147, ST258, and ST307, ST290 has been reported infrequently. Nevertheless, NDM-5-producing ST290 has been associated with nosocomial outbreaks in China, suggesting that this lineage has the potential for clonal dissemination [23]. Therefore, the identification of an NDM-5-producing ST290 isolate in the present case is epidemiologically noteworthy, despite the absence of overseas exposure. Furthermore, the whole-genome sequence reported here contributes to the limited molecular epidemiological data on NDM-5-producing *K. pneumoniae* in Japan. Notably, the patient had no previous exposure to therapeutic antibiotics during chemotherapy, except for prophylactic TMP-SMX, suggesting that direct antimicrobial selection pressure was unlikely to contribute to the acquisition of this isolate. This finding raises the possibility that acquisition occurred independently of recent broad-spectrum antibiotic exposure.

Currently, NDM-5 is one of the predominant carbapenemases in neighboring countries such as China, Taiwan, and South Korea [10-12]. In China, the rapid expansion of high-risk clones of E. coli ST167, ST410, and ST648 has been reported [24,25]. Given the increasing movement of people and goods between Japan and neighboring East Asian countries, where NDM-5 is prevalent, the repeated introduction of NDM-5-producing organisms into Japan is likely to have contributed to the evolving molecular epidemiology of CPE. In recent years, NDM-5-carrying bacteria have been detected in environmental samples, such as wastewater, in Japan, suggesting that NDM-5-producing organisms or blaNDM-5-carrying plasmids may be present in environmental reservoirs [26,27]. Although this single case does not establish community dissemination, it raises the possibility of domestic acquisition of NDM-5-producing organisms and underscores the need for continued molecular surveillance to clarify the transmission dynamics in Japan.

Furthermore, the Infectious Diseases Society of America (IDSA) guidelines recommend a combination of aztreonam (AZT) and ceftazidime/avibactam (CAZ/AVI) or cefiderocol (CFDC) for the treatment of NDM-5 [2]. However, recent reports have shown that NDM-5 strains acquire resistance to CFDC through concurrent mutations in CirA and PBP3 [28]. The isolate in the present case exhibited an MIC of CFDC at the upper limit of the susceptible range, suggesting that susceptibility testing should guide the use of CFDC, particularly in regions where NDM-5-producing isolates are emerging.

## Conclusions

This case describes a urinary tract infection caused by NDM-5-producing *K. pneumoniae* ST290 in a young patient without a history of overseas travel or exposure to foreign healthcare facilities. Whole-genome sequencing identified an NDM-5-producing ST290 isolate, contributing to the limited molecular epidemiological data on this lineage in Japan. Although this single case does not establish community dissemination, it raises the possibility of domestic acquisition of NDM-5-producing Enterobacterales. These findings highlight the importance of considering CPE, including NDM-producing isolates, when highly carbapenem-resistant Enterobacterales are encountered, even in patients without overseas exposure. Continued molecular surveillance and appropriate infection control measures are warranted to monitor and prevent further dissemination of non-IMP-type CPE in Japan.
